# Closing Editorial: Advances in Chitin and Chitosan-Based Materials: Preparation and Applications

**DOI:** 10.3390/polym17152060

**Published:** 2025-07-28

**Authors:** Xianzhi Kong, Dawei Zhang

**Affiliations:** 1 Institute of Petrochemistry, Heilongjiang Academy of Sciences, Harbin 150040, China; 2 State Key Laboratory of Woody Oil Resources Utilization, Northeast Forestry University, Harbin 150040, China

## 1. Introduction

Chitin and chitosan-based materials are widely used and researched in healthcare, pharmaceutical, biomedical engineering, and related fields due to their biological activity. This Special Issue focuses on the preparation and application of materials based on chitin and chitosan, with the aim of presenting the latest research advances in biomedical applications and highlighting the potential for further innovative research. This Special Issue showcases the potential of chitin and chitosan-based materials by examining innovative approaches to maximize their structural, physical–chemical, and bioactive qualities.

## 2. An Overview of the Published Articles

The Special Issue highlights the potential uses of chitin and chitosan-based materials in biomedical, pharmaceutical, and sustainable practices and related fields. Liao et al. prepared a class of multifunctional chitosan-based hydrogels through dual cross-linking with borate esters and hydrogen bonds, achieving glucose-sensitive controlled release, providing a new method for the design of polysaccharide-based hydrogels [1]. Zhou et al. designed a flexible, low-cost, biomimetic spiral hollow bacterial cellulose-chitosan fibre, which could be an attractive candidate to replace other petroleum-based sutures [2].

Li et al. developed an emulsion carrying thyme essential oil via inducing cross-linking of chitosan particles through hydrogen bonds and electrostatic interactions. This emulsion has great potential for prolonging the storage life of strawberries [3]. Shi et al. prepared a hydrogel using chitosan and coumarin as raw materials for the controlled release of taxifolin [4]. Zhang et al. prepared chitosan-based Janus nanofiber membranes as wound dressings, which have vast potential applications in skin tissue engineering [5]. Blanzeanu et al. used extrusion moulding to prepare composite blends of chitosan, providing a sustainability–recycling-based approach to converting seafood waste into cutting-edge functional materials [6].

Li et al. summarize the state-of-the-art developments in chitosan-based dressing materials, highlighting the benefits in burn–wound treatment and examining the key challenges and potential future directions for chitosan-based dressing materials. This mini-review provides a new viewpoint on the evolution of wound dressings for burn care [7]. 

Zhou et al. provide a broad overview of chitosan extraction and modification technologies, with a focus on their applications in environment, energy, and biomedicine. It uses a novel classification framework to provide readers with the most detailed analysis for a systematic understanding of the latest research progress [8].

Together, these eight articles showcase the versatility of chitosan materials and discuss potential preparations and applications, touching upon the variety of ways in which they could be used in many fields, ranging from environmental sustainability to medical therapeutics.

## 3. Conclusions

The breadth of chitosan-based technologies and their potential to solve practical problems are highlighted in this Special Issue. From sophisticated biomedical systems to ecofriendly preservation solutions, together, these studies emphasize the versatility, biodegradability, and efficacy chitin and chitosan. As research progresses, further optimization of chitin and chitosan properties may result in more relevant and effective applications in industrial and clinical settings.
